# Chromosomal abnormality variation detected by G‐banding is associated with prognosis of diffuse large B‐cell lymphoma treated by R‐CHOP‐based therapy

**DOI:** 10.1002/cam4.1342

**Published:** 2018-02-23

**Authors:** Yoshimi Mizuno, Taku Tsukamoto, Eri Kawata, Nobuhiko Uoshima, Hitoji Uchiyama, Isao Yokota, Saori Maegawa, Tomoko Takimoto, Kazuna Tanba, Yayoi Matsumura‐Kimoto, Saeko Kuwahara‐Ota, Yuto Fujibayashi, Mio Yamamoto‐Sugitani, Yoshiaki Chinen, Yuji Shimura, Shigeo Horiike, Masafumi Taniwaki, Tsutomu Kobayashi, Junya Kuroda

**Affiliations:** ^1^ Division of Hematology and Oncology Department of Medicine Kyoto Prefectural University of Medicine Kyoto Japan; ^2^ Department of Hematology Japanese Red Cross Kyoto Daini Hospital Kyoto Japan; ^3^ Department of Hematology Japanese Red Cross Kyoto Daiichi Hospital Kyoto Japan; ^4^ Department of Biostatistics Kyoto Prefectural University of Medicine Kyoto Japan

**Keywords:** Chromosomal abnormality, chromosomal abnormality variations, diffuse large B‐cell lymphoma, karyotypic evolution

## Abstract

Diffuse large B‐cell lymphoma (DLBCL), which is the most prevalent disease subtype of non‐Hodgkin lymphoma, is highly heterogeneous in terms of cytogenetic and molecular features. This study retrospectively investigated the clinical impact of G‐banding‐defined chromosomal abnormality on treatment outcomes of DLBCL in the era of rituximab‐containing immunochemotherapy. Of 181 patients who were diagnosed with DLBCL and treated with R‐CHOP or an R‐CHOP‐like regimen between January 2006 and April 2014, metaphase spreads were evaluable for G‐banding in 120. In these 120 patients, 40 were found to harbor a single chromosomal aberration type; 63 showed chromosomal abnormality variations (CAVs), which are defined by the presence of different types of chromosomal abnormalities in G‐banding, including 19 with two CAVs and 44 with ≥3 CAVs; and 17 had normal karyotypes. No specific chromosomal break point or numerical abnormality was associated with overall survival (OS) or progression‐free survival (PFS), but the presence of ≥3 CAVs was significantly associated with inferior OS rates (hazard ratio (HR): 2.222, 95% confidence interval (CI): 1.056–4.677, *P *=* *0.031) and tended to be associated with shorter PFS (HR: 1.796, 95% CI: 0.965–3.344, *P *=* *0.061). In addition, ≥3 CAVs more frequently accumulated in high‐risk patients, as defined by several conventional prognostic indices, such as the revised International Prognostic Index. In conclusion, our results suggest that the emergence of more CAVs, especially ≥3, based on chromosomal instability underlies the development of high‐risk disease features and a poor prognosis in DLBCL.

## Introduction

Non‐Hodgkin lymphoma (NHL) is a highly prevalent hematologic malignancy with a variety of disease subtypes with different histological findings, etiology, molecular features, and clinical manifestations. Diffuse large B‐cell lymphoma (DLBCL) is an aggressive and the most frequent subtype of NHL that is defined by histological characterization of diffuse and unstructured sheet architectures of medium‐ to large‐sized abnormal B‐cell lineage lymphoid cells 1. Recent progress in immunochemotherapy combining rituximab, an anti‐CD20 monoclonal antibody, and cytotoxic agents has greatly improved the treatment outcome of DLBCL, but approximately 30% of patients with DLBCL remain incurable 2, 3.

Chromosome abnormalities play critical roles in emergence of cancer‐initiating cells and in disease progression of most cancers. Especially in hematologic malignancies, identification of disease‐specific chromosomal abnormalities is essential for differential diagnosis of molecularly or biologically distinct disease subtypes with significantly different prognoses 4. In B‐cell NHLs, disease subtype‐specific chromosomal abnormalities are strongly associated with disease development, such as translocation t(14;18) involving *BCL2* gene rearrangement in follicular lymphoma 5, 6, t(11;14) involving *Cyclin D1* (*CCND1*) gene rearrangement in mantle cell lymphoma 7, or t(8;14) involving *c‐MYC* gene rearrangement in Burkitt lymphoma 8, 9. However, no specific chromosomal aberration has been shown to be diagnostically or prognostically relevant in DLBCL, although several abnormalities have been repeatedly identified. Double‐hit or triple‐hit B‐cell lymphomas harboring concomitant chromosomal rearrangements involving *c‐MYC* and *BCL2* and/or *BCL6* genes with unfavorable prognoses have previously been included in DLBCL, but these are considered to be an independent disease subtype in the latest WHO classification updated in 2016 10.

Tumors cells of DLBCL frequently possess random and complex chromosomal abnormalities and sometimes exhibit more than two chromosomal abnormality variations (CAVs), such as karyotypic evolution with additional chromosomal abnormalities or totally different patterns of chromosomal abnormalities. This suggests a contribution of karyotypic/genetic instability and additional acquisition of genetic changes to tumor progression. Considering that acquisition of additional karyotypic/genetic changes is vertically transmittable mechanisms for cancer adaptation and progression by constructing intratumor heterogeneity, which eventually leads to acquisition of therapeutic resistance 11, and in this study, we retrospectively investigated the clinical effects of particular chromosomal rearrangements and the number of CAVs on clinical outcomes of patients with DLBCL treated by rituximab, cyclophosphamide, doxorubicin, vincristine, and prednisolone (R‐CHOP)‐based chemotherapy in a real‐world clinical setting.

## Materials and Methods

### Patients

We retrospectively analyzed the medical records of 465 patients with DLBCL diagnosed at three independent institutes in Kyoto, Japan, between January 2006 and April 2014. Among these patients, those with karyotypic analyses of biopsied specimens performed by G‐banding before the start of treatment by R‐CHOP or with an R‐CHOP‐like regimen were included in this study. The R‐CHOP‐like regimens included reduced R‐CHOP, R‐pirarubicin (THP)‐COP, and these chemotherapies combined with radiotherapy. This study was conducted in accordance with the ethical principles of the Declaration of Helsinki and was approved by the institutional review boards of all participating institutes.

### Karyotypic analysis and counting of chromosomal abnormality variations (CAVs)

Classic karyotyping of metaphases by G‐banding was performed as described elsewhere 12. To avoid bias, interphase fluorescence in situ hybridization and molecular diagnostic tests were not considered for this analysis. Twenty metaphase spreads were normally analyzed for one biopsied specimen, and karyotypic aberration was determined in accordance with the International System for Human Cytogenetic Nomenclature (ISCN); however, the number of evaluable tumor‐derived metaphase cells for karyotypic analysis was <20 in some patients.

The number of CAVs was counted as follows: (i) 1, in a case with only one pattern of chromosomal abnormality identified throughout all analyzed metaphase cells, (ii) 2, in a case with metaphase cells with a major pattern of chromosomal aberration and a minor additional pattern of chromosomal aberration, (iii) also 2, in a case with metaphase cells with a major pattern of chromosomal aberration and a totally different pattern of chromosomal aberration, (iv) ≥3, in a case with metaphase cells with a major pattern of chromosomal aberration and more than two different patterns of additional chromosomal aberration, (v) also ≥3, in a case with more than three patterns of metaphase cells with totally different patterns of chromosomal aberration, and (vi) 0, in a case in which only a normal karyotype was identified. Constitutional karyotypes were not counted as abnormal. The counts for (ii) to (v) were used even if the findings were observed only in one metaphase spread.

**Figure 1:**
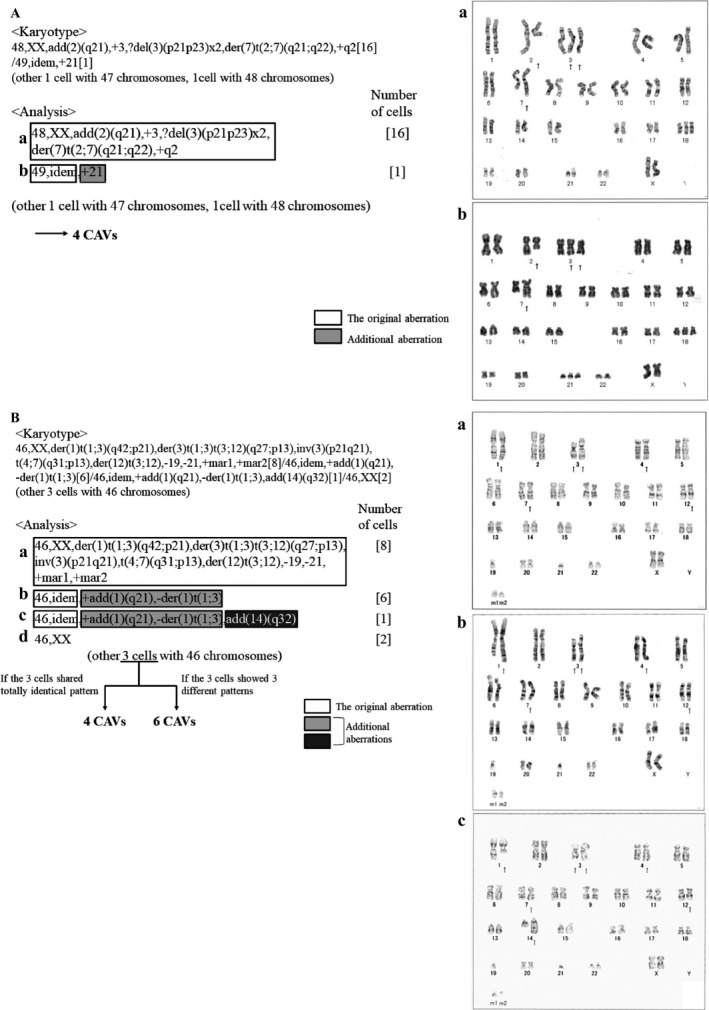
Two examples of the way how we defined the number of CAVs. White squares represent the original aberrations (a), and light and dark gray squares represent additional aberrations. b, c, and d represent minor clones with additional abnormalities and/or different chromosomal feature.

To show examples of how we defined the number of CAVs, G‐banding data from two patients with DLBCL are shown in Figure 1A and B. As the first example, chromosomal feature of the patient #1 in Table S1 is shown in Figure 1A. The figure presents a major pattern of chromosomal aberration defined “a” found in 16 metaphase spreads, a minor pattern of additional chromosomal aberration defined “b” found in one metaphase spread, and other two metaphase spreads with 47 and 48 chromosomes. As the result, this case presents four CAVs with “a,” “b,” and the two unknown patterns. As the second example, chromosomal feature of the patient #29 in Table S1, a more complicated case, is shown in Figure 1B. The figure presents a major pattern of chromosomal aberration defined “a” found in eight metaphase spreads, two minor additional patterns of chromosomal aberration defined “b” and “c” found in six and one metaphase spreads respectively, two metaphase spreads defined “d” with no chromosomal aberration, and three other metaphase spreads with 46 chromosomes showing chromosomal aberrations, but further details are unknown. In this case, on the assumption that all the three metaphase spreads with unknown details shared totally identical pattern of chromosomal aberration, the number of CAVs would be 4 with “a,” “b,” “c,” and the unknown pattern. On the assumption that the three metaphase spreads with unknown details showed three different patterns of chromosomal aberration, the number of CAVs would be 6 with “a,” “b,” “c,” and three unknown patterns. In brief, the number of CAVs is 4 or more and 6 or less, as far as we can confirm from the available data. In contrast to the definition of clones by ISCN, requiring a chromosome gain or a structural rearrangement to be present in least two cells and a loss of chromosome to be present in at least three cells to be accepted as clonal 13, our definition of CAV includes single cell abnormalities such as the pattern “b” of the patient #1 or the pattern “c” of the patient #29.

**Figure 2:**
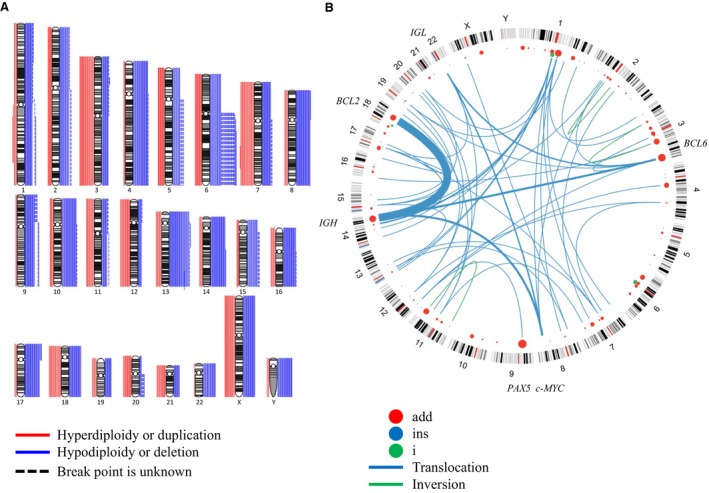
Numerical chromosomal abnormalities and chromosomal rearrangement break points/translocations. (A) Numerical abnormalities. Red lines on the left of each karyogram indicate gain or hyperdiploidy, and blue lines on the right indicate loss or hypodiploidy. In cases in which precise break points were not identified, dashed lines are shown. The number of lines for each chromosome shows the number of tumors with the abnormality. The frequent gains were +3 (*N* = 19), +7 (*N* = 18), and +18 (*N* = 16), and the frequent losses were −13 (*N* = 27), −14 (*N* = 20), −4 (*N* = 20), −8 (*N* = 19), and −10 (*N* = 20). (B) Structural abnormalities. Red points indicate break points of additional materials of unknown origins. The size of each point shows the number of tumors. Blue lines are chromosomal translocations. Each line weight shows the number of tumors. Abnormalities detected at a rate of >5.0% included chromosomal rearrangements involving 3q27 (*N* = 16), 7q22 (*N* = 7), 8q24 (*N* = 8), 9p13 (*N* = 11), 11q13 (*N* = 6), 14q32 (*N* = 29), and 18q21 (*N* = 20).

### Statistics

Overall survival (OS) was defined as the time from start of treatment to death from any cause. Progression‐free survival (PFS) was defined as the time from start of treatment to the first sign of progression or death from any cause. PFS and OS rates were estimated using the Kaplan–Meier method. A log‐rank test and Cox proportional hazards regression analysis were used to evaluate differences between number of CAVs (≥3/0‐2) in OS and PFS. We also adjusted clinical background factors as confounders by Cox proportional hazard regression. Relationships of the number of CAVs with clinical background factors and prognostic indices: the International Prognostic Index (IPI) 14, revised IPI (R‐IPI) 15, National Comprehensive Cancer Network (NCCN)‐IPI 16, and Kyoto Prognostic Index (KPI), which we have recently developed 17, were evaluated by chi‐square test, except for that with age, which was examined by *t*‐test.

## Results

### Patients

Among 465 reviewed patients, karyotypic analyses by G‐banding were performed on biopsied tumor specimens before the start of R‐CHOP or R‐CHOP‐like regimens in 181. As shown in Table 1, the median age of the 181 patients was 70 years old, and the rates of male patients and patients with Eastern Cooperative Oncology Group performance status (PS) worse than 2 were 49.2% and 19.3%, respectively. The PFS and OS rates at 3 years of 181 patients were 68.7% and 79.7%, respectively. All three prognostic indices used in the study (R‐IPI, NCCN‐IPI, and KPI) largely successfully stratified the risks of patients, and survival of the KPI high‐risk group was the poorest in our analysis (Fig. S1).

**Table 1:** Clinical background of the patients

Item	Value
Age, median (range)	70 (34–88)
Gender	
Male, *n* (%)	89 (49.2)
Female, *n* (%)	92 (50.8)
Performance status, *n* (%)	
0–1	146 (80.7)
≥2	35 (19.3)
Ann Arbor‐defined disease stage, *n* (%)	
Limited	84 (46.4)
Advanced	97 (53.6)
Serum LDH level, *n* (%)	
Normal range	75 (41.4)
> x1–3 UNL	86 (47.5)
≥ x3 UNL	20 (11.0)
3‐year PFS (%)	68.7
3‐year OS (%)	79.7
R‐IPI, *n* (%)	
Very Good	76 (12.3)
Good	24 (38.9)
Poor	81 (48.8)
NCCN‐IPI, *n* (%)	
Low	22 (10.8)
Low‐Int	69 (35.5)
High‐Int	59 (34.5)
High	31 (19.2)
KPI, *n* (%)	
Low	71 (36.0)
Low‐Int	70 (38.9)
High‐Int	15 (9.4)
High	25 (15.8)

PFS, progression‐free survival; OS, overall survival; R‐IPI, revised International Prognostic Index; NCCN‐IPI, National Comprehensive Cancer Network‐IPI; KPI, Kyoto Prognostic Index.

### Results of chromosomal analysis

Among the 181 patients with biopsied specimens subjected to G‐banding, metaphase spreads were available for karyotypic analysis by G‐banding in 120 patients, and not available in 61 patients (Tables 2 and S1, Fig. S2). Neither OS nor PFS differed significantly between these groups of patients (Fig. S3). In the 120 patients with available metaphase spreads, 103 and 17 had abnormal and normal karyotypes, respectively. Regarding structural chromosomal abnormalities, 14q32 rearrangements were identified in 26 patients (21.7%), and other abnormalities detected at a rate of ≥5.0% included chromosomal rearrangements involving 3q27, 7q22, 8q24, 9p13, 11q13, and 18q21, which were identified in 16 (13.3%), 7 (5.8%), 8 (6.7%), 11 (9.2%), 6 (5.0%), and 20 (16.7%) cases, respectively. Numerous numerical chromosomal abnormalities were also detected, including both chromosomal gain and loss: The most frequent gains were +3 (*N* = 19, 15.8%), +7 (*N* = 18, 15.0%), and +18 (*N* = 16, 13.3%), and the most frequent losses were −13 (*N* = 27, 22.5%), −14 (*N* = 20, 16.7%), −4 (*N* = 20, 16.7%), −8 (*N* = 19, 15.8%), and −10 (*N* = 20, 16.7%) (Fig. 2). Marker chromosomes were also frequently observed. In the 103 patients with abnormal karyotypes, 40 harbored a single type chromosomal aberration only (i.e., one CAV), 19 had two CAVs, and 44 had ≥3 CAVs (Table S1).

**Table 2:** Comparison between patients with ≥3 chromosomal abnormality variations (CAVs) and 0–2 CAVs in 120 patients with available metaphase spreads

Subject	Total	CAV 0–2	CAV ≥3	*P*
Patient number	120	76	44	
Age, median (range)	67.7 (34–85)	67.8	67.6	0.917
Gender (*n*)				
Male	63	40	23	1.000
Female	57	36	21	
Performance status (*n*)				
0–1	92	65	27	0.005
≥2	28	11	17	
Ann Arbor‐defined disease stage (*n*)				
Limited	50	34	16	0.481
Advanced	70	42	28	
Serum LDH level (*n*)				
Normal range	50	35	15	0.378
> x1–3 UNL	53	32	21	
≥ x3 UNL	17	9	8	
Extranodal involvement (*n*)				
None	59	39	20	0.668
Present	61	37	24	
R‐IPI (*n*)				
Very Good/Good	60	45	15	0.014
Poor	60	31	29	
NCCN‐IPI (*n*)				
Low/Low‐Int/High‐Int	95	64	31	0.120
High	25	12	13	
KPI (*n*)				
Low/Low‐Int/High‐Int	98	67	31	0.030
High	22	9	13	

The presence of ≥3 CAVs is shown to have significant association with PS, OS, R‐IPI, and KPI, but no association with other clinical backgrounds.

### Prognostic impacts of sites of chromosomal rearrangements and number of CAVs

**Figure 3:**
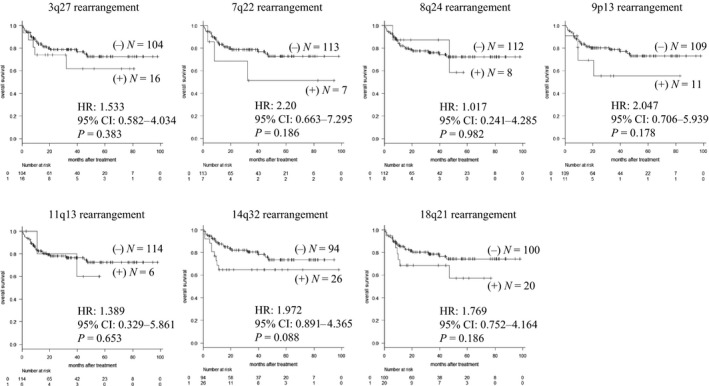
Overall survival of patients with and without chromosomal rearrangements involving 3q27, 7q22, 8q24, 9p13, 11q13, 14q32, and 18q21. HR, hazard ratio; CI, confidence interval. None of the sites of chromosomal rearrangement was significantly associated with OS.

**Figure 4:**
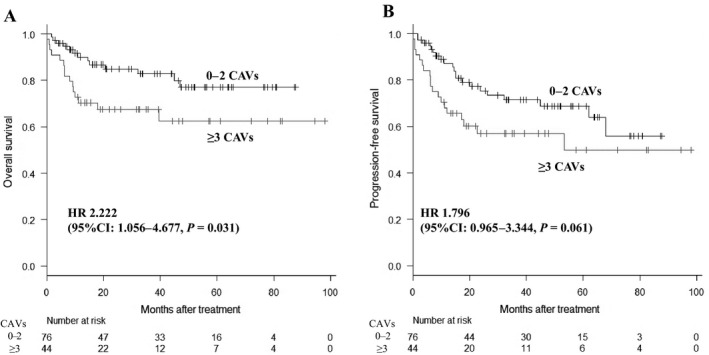
Overall survival (A) and progression‐free survival (B) of patients with 0–2 and ≥3 CAVs. HR, hazard ratio; CI, confidence interval. Cases with ≥3 CAVs had significantly poorer 3‐year OS compared to those with 0–2 CAVs (67.6% vs. 82.8%, *P* = 0.031) and tended to have shorter PFS.

In investigations of the impact of sites of chromosomal rearrangements on survival outcomes, though the rearrangements involving 3q27, 7q22, 8q24, 9p13, 11q13, 14q32, or 18q21 tended to show elevated hazard ratio (HR) for OS, the statistical significance was not made clear (Fig. 3). In contrast, cases with ≥3 CAVs had significantly poorer OS compared to those with 0–2 CAVs (HR: 2.222, 95% confidence interval (CI): 1.056–4.677, *P *=* *0.031) (Fig. 4A), and tended to have shorter PFS (HR: 1.796, 95% CI: 0.965–3.344, *P *=* *0.061) (Fig. 4B). OS and PFS did not differ significantly between patients with one CAV and those without a CAV (data not shown). To adjust confounding, we next performed multivariate analysis including ≥3 CAVs with age and gender, which are obviously clear of influence by the number of CAVs. Presence of ≥3 CAVs showed significant impact to OS (HR: 2.142, 95% CI: 1.016–4.515, *P *=* *0.045) and tended to show negative influence to PFS (HR: 1.768, 95% CI: 0.949–3.292, *P *=* *0.072). We also performed multivariate analysis including ≥3 CAVs, age, gender, stage, and extranodal involvement, and the presence of ≥3 CAVs still showed relation to elevated hazard ratio for OS (HR: 2.066, 95% CI: 0.975–4.377, *P *=* *0.058) and PFS (HR: 1.718, 95% CI: 0.921–3.207, *P *=* *0.089). We did not regard the rest of prognostic factors utilized in R‐IPI, NCCN‐IPI, and KPI (poor performance status, elevated lactate dehydrogenase (LDH), and decreased albumin) as confounders, because it was unlikely that these factors affect the number of CAVs.

### Relationships among background factors, prognostic indexes, and number of CAVs

An evaluation of relationships between background factors and the number of CAVs showed that only PS ≥2 was significantly associated with ≥3 CAVs. Age, gender, serum LDH level, extranodal involvement, and Ann Arbor disease stage were not associated with the number of CAVs. In risk stratification using prognostic indices for DLBCL, patients with ≥3 CAVs were significantly more frequently found to be at high risk using the R‐IPI and KPI (Table 2). In each prognostic index‐defined risk group, the number of CAVs was not significantly associated with different survival outcomes of patients (data not shown).

## Discussion

Acquisition of additional chromosomal abnormality (i.e., karyotypic evolution) is fueled by chromosomal instability due to loss of chromosome fidelity 18 and is frequently observed in hematologic malignancies in daily clinical practice. The prognostic impact of karyotypic evolution differs depending on the type of hematologic malignancy. For instance, it is associated with a poor prognosis in myelodysplastic syndrome 19, but does not significantly influence the prognosis of acute leukemias with core‐binding factor translocations or with *t*(9;11) 20. In DLBCL, the clinical impact of karyotypic evolution has not been fully evaluated, while the failure of anaphase accomplishment has been associated with a poor prognosis 21.

This study shows that chromosomal abnormality with ≥3 CAVs is related to a poor prognosis in DLBCL. An increased number of CAVs might be led by chromosomal instability and cause more advanced karyotypic evolution. The number of CAVs was also correlated with prognostic indices based on background factors and laboratory findings. Thus, our findings reveal that high‐risk patients defined by R‐IPI or KPI have distinct cytogenetic instability, compared to patients stratified as lower risk by these indices. These results suggest a need for identification of molecular mechanisms underlying chromosome instability in high‐risk patients. Our study showed the positive association between ≥3 CAVs and worse prognosis, while one or two CAVs did not show prognostic impact in DLBCL. Although the precise mechanism underlying the different prognostic impact between ≥3 CAVs and 1/2 CAVs remained to be verified, we suspect that ≥3 CAVs surrogates the boundary of chromosome instability which apparently makes the clinical manifestation more progressive with R‐CHOP(‐like) therapy.

In G‐banding analysis used in daily clinical practice, we found that the number of CAVs is often unclear, especially when there are many different CAVs to analyze. This problem was the major limitation in this study. However, even in this situation, it was possible to divide DLBCL into cases with 0, 1, 2, and ≥3 CAVs. We eventually focused on comparison of DLBCL with 0–2 and ≥3 CAVs because OS was most markedly different between these two groups. Another limitation was that metaphase spreads from G‐banding were not available in about one‐third of biopsied DLBCL specimens. Because our study suggests that increased number of CAVs due to chromosomal instability contribute to the development of high‐risk disease feature in DLBCL, our next research is focusing on the development of novel method which enables the detection of CAVs or chromosomal instability using nondividing cells and thereby provides a clue for therapeutic choice which is available in all patients with DLBCL in future.

Previous studies have evaluated the prognostic impacts of cytogenetic and molecular abnormalities in DLBCL. Although double‐hit or triple‐hit B‐cell lymphomas are well‐known with worse prognosis, previous studies concerning the prognostic impact of a *c‐MYC* rearrangement alone have been controversial. Some studies demonstrated that a *c‐MYC* rearrangement leads to poor prognosis even without *BCL2* or *BCL6* rearrangements 22, 23, while a *c‐MYC* rearrangement alone did not show significant prognostic impact in other studies 24, 25. The rearrangement of *BCL2*,* BCL6,* or *PAX5* alone has not been associated with worse outcome in DLBCL patients 26, 27, while the prognostic impact of *CCND1* rearrangement in DLBCL has been controversial 28. In this study, we found no significant association between the OS and chromosomal rearrangements involving 8q24, the site where *c‐MYC* locates, as well as rearrangements at other break points, including 3q27 involving *BCL6*, 9p13 involving *PAX5*, 11q13 involving *CCND1,* and 18q21 involving *BCL2*. While our data did not clarify the prognostic impact of the chromosomal rearrangements at specific break points, the number of CAVs showed significant association to the OS.

With recent advanced genomic technologies, such as gene expression profiling (GEP), microRNA (miRNA) profiling, genome‐wide copy number abnormalities, global methylation, mutation spectrum, and whole‐genome sequencing (WGS), more detailed molecular changes have been identified in DLBCL. The validity of GEP classification which reflects the cell‐of‐origin of tumor cells, that is, germinal center B‐cell‐like (GCB) type and activated B‐cell‐like (ABC) type 29, is supported by other study with high‐resolution genome‐wide copy number analysis 30. In addition, gene methylation profiles and miRNA signatures have been reported to be different between GCB‐DLBCL and ABC‐DLBCL 31, 32. WGS has identified widespread genomic mutations/rearrangements which are involved in lymphomagenesis of DLBCL 33. Concerning the genomic instability of DLBCL, targeted sequencing of 73 key DNA repair genes discovered somatic alterations in several novel and/or potentially functional important mutation targets in DLBCL, including *CHEK2*,* PARP1,* and *DDB1*, and several nonhomologous end‐joining (NHEJ) genes (*DLRE1C*,* PRKDC*,* XRCC5,* and *XRCC6*), as well as mismatch repair (MMR) genes (*EXO1*,* MSH2,* and *MSH6*) 34. Somatic mutations in DNA repair genes affected approximately half of DLBCL cases analyzed, and mutations in subsets of these genes, especially those belonging to the MMR and NHEJ pathways, indeed associated with different forms of genetic instability in tumors. However, those advanced genomic technologies have not been routinely used in daily clinical practice for DLBCL due to its technical difficulty and high cost. We, in this study, propose the relation of the number of CAVs to the prognosis of DLBCL which can be easily determined from routinely performed G‐banding. Future studies are necessary to explore the relationships among the number of CAVs, GEP classification, and gene mutation profiles for genetic instability in DLBCL.

Compared with GCB‐DLBCL, ABC‐DLBCL has been associated with an inferior prognosis even with rituximab‐containing immunochemotherapy, while recent studies have shown that strategies using lenalidomide, an immunomodulatory drug, or targeting B‐cell receptor signal improve the treatment outcome of ABC‐DLBCL 35, 36. Considering that difference in biological character between ABC‐DLBCL and GCB‐DLBCL is based on the distinct role of BCR signal pathway which is a completely different perspective from chromosomal instability, we expect patients with ≥3 CAVs to be found in both ABC and GCB‐DLBCL groups. However, it is possible that the clinical impact of the number of CAVs differs between ABC and GCB‐DLBCL, which is the remaining question we next have to work on.

In conclusion, this study suggests that more advanced cytogenetic evolution reflected by more CAVs is related to development of high‐risk disease and poor prognosis in DLBCL under R‐CHOP‐like strategy. The molecular basis for chromosomal instability requires further studies for identification of a high‐risk biomarker and development of novel diagnostic method for chromosomal instability and targeted therapeutics.

## Conflict of Interest

The authors have declared no conflict of interest.

## Supporting information


**Figure S1.** Overall survival of patients classified by R‐IPI, NCCN‐IPI, and KPI.

**Figure S2.** Patient cohort selection.

**Figure S3.** Overall survival (A) and progression‐free survival (B) of patients with and without available metaphase spreads.

**Table S1.** Clinical features, chromosomal abnormalities and number of chromosomal abnormality variations (CAVs) in 120 DLBCL patients with available metaphase spreads.
