# Supplementary figures and images for: Chromosomal abnormality variation detected by G‐banding is associated with prognosis of diffuse large B‐cell lymphoma treated by R‐CHOP‐based therapy
Source: Cancer Med. 2018 Feb 23;7(3):655–64. doi: 10.1002/cam4.1342 (PMC5852349; doi:10.1002/cam4.1342)

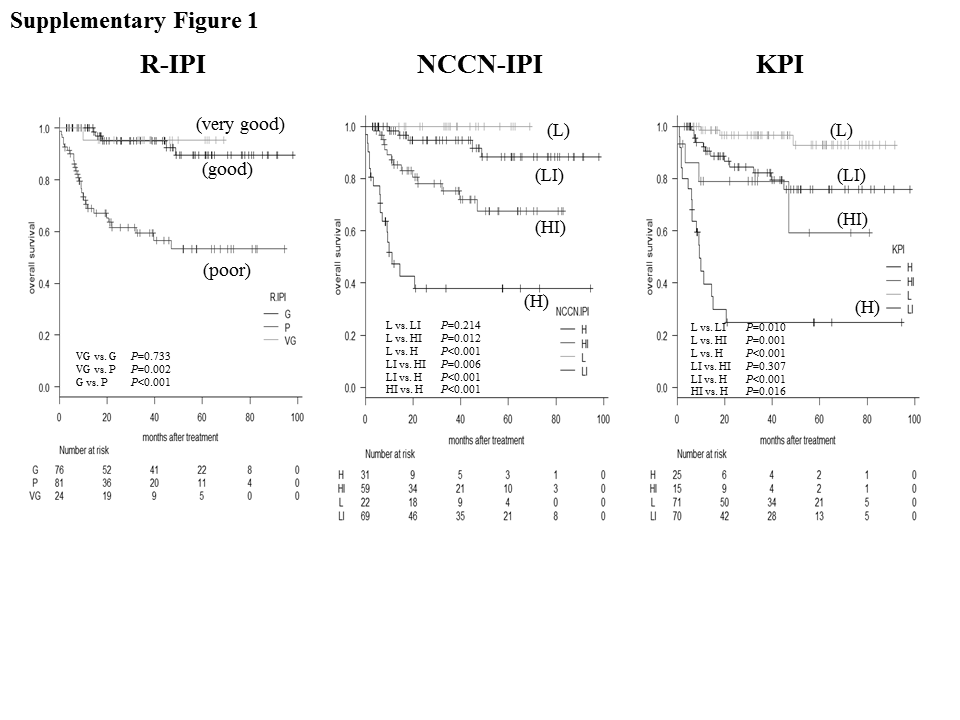

Supplement: Supplementary file 1 — Figure S1. Overall survival of patients classified by R‐IPI, NCCN‐IPI, and KPI. [file CAM4-7-655-s001.tif]

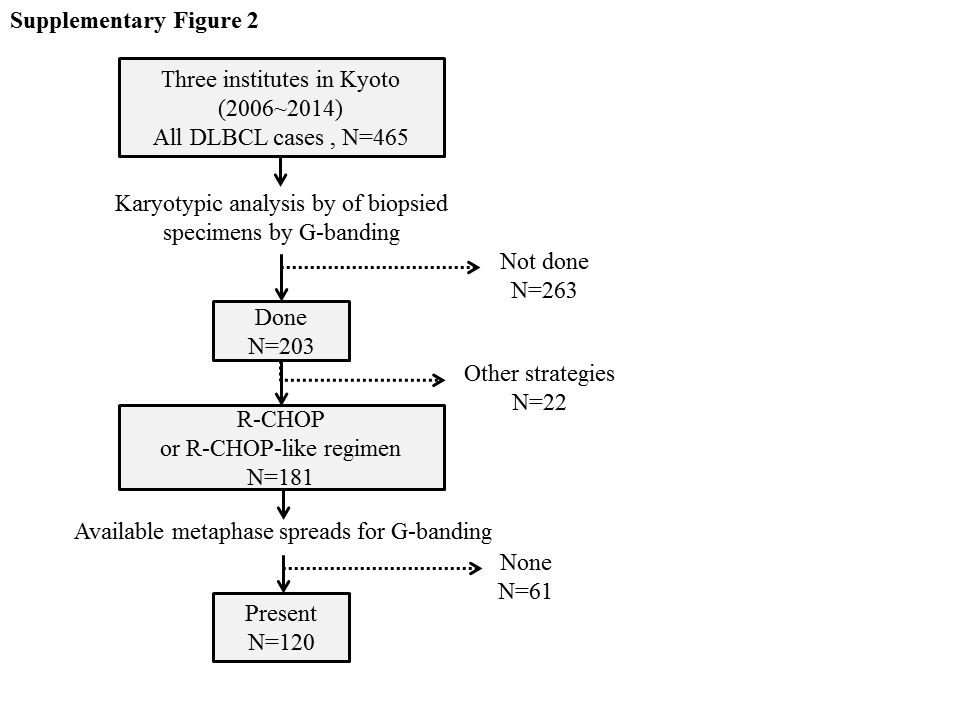

Supplement: Supplementary file 2 — Figure S2. Patient cohort selection. [file CAM4-7-655-s002.tif]

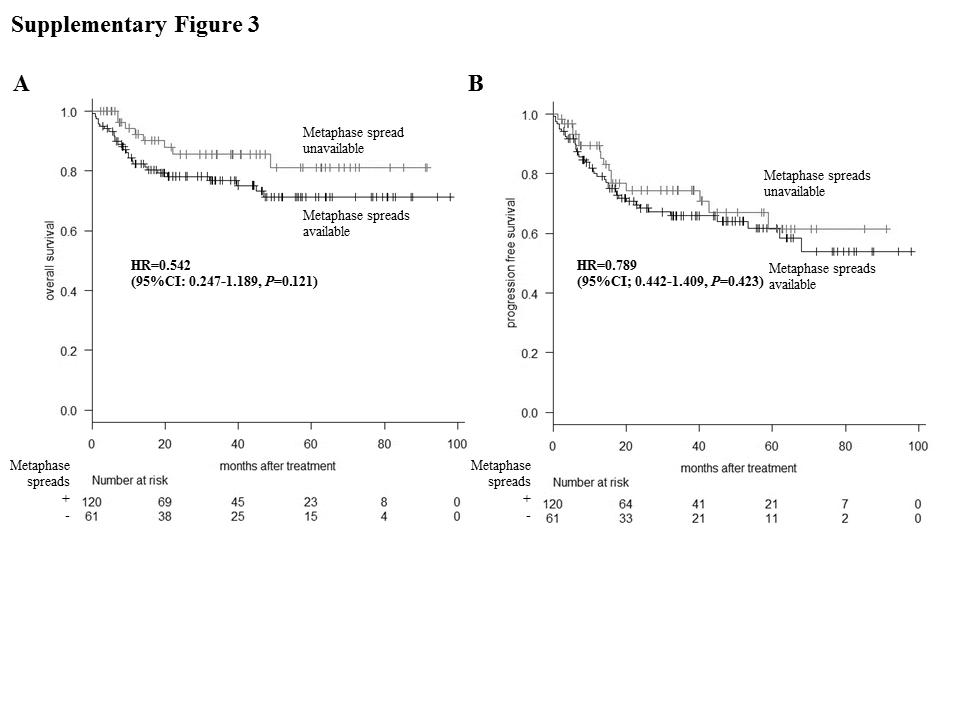

Supplement: Supplementary file 3 — Figure S3. Overall survival (A) and progression‐free survival (B) of patients with and without available metaphase spreads. [file CAM4-7-655-s003.tif]
