# Supplementary material for: Chromosomal abnormality variation detected by G‐banding is associated with prognosis of diffuse large B‐cell lymphoma treated by R‐CHOP‐based therapy
Source: Cancer Med. 2018 Feb 23;7(3):655–64. doi: 10.1002/cam4.1342 (PMC5852349; doi:10.1002/cam4.1342)
Supplement: Supplementary file 4 — Table S1. Clinical features, chromosomal abnormalities and number of chromosomal abnormality variations (CAVs) in 120 DLBCL patients with available metaphase spreads. [file CAM4-7-655-s004.docx]

| Supplementary Table 1 Clinical features, chromosomal abnormalities and the number of chromosomal abnormality variations (CAVs) of 120 DLBCL patients with available metaphase spreads in this study. | | | | | | | | | | | | | | | |
| --- | --- | --- | --- | --- | --- | --- | --- | --- | --- | --- | --- | --- | --- | --- | --- |
| No | Age | Sex | IPI | R-IPI | NCCN  -IPI | KPI | G-band | 14q32 | 3q27 | 7q22 | 8q24 | 9p13 | 11q13 | 18q21 | CAVs |
| 1 | 72 | F | H | P | H | H | 48,XX,add(2)(q21),+3,?del(3)(p21p23)x2,der(7)t(2;7)(q21;q22),+q2[16]/49,idem,+21[1] (other 1 cell with 47 chromosomes, 1cell with 48 chromosomes) |  |  | + |  |  |  |  | ≧3 |
| 2 | 66 | F | H | P | H | H | 46,XX,del(6)(q13),add(9)(p13),-13,add(14)(q32),add(16)(p11),+mar[11]/46,XX,add(12)(p13),add(17)(q21)[1]/46,XX[8] | + |  |  |  | + |  |  | 2 |
| 3 | 68 | F | L | G | LI | LI | 46,XX,+3,add(6)(q21),-9,add(12)(p11),-14,-17,+18,-22,+mar1,+mar2[4] |  |  |  |  |  |  |  | 1 |
| 4 | 75 | F | LI | G | LI | L | 47,X,-X,t(3;3)(q12;q27),+del(5)(q31q33),i(6)(p10),der(8)t(X;8)(p11.2;p21),+10,add(22)(q13)[9]/46,XX[10] (other 1 cell with 48 chromosomes) |  | + |  |  |  |  |  | 2 |
| 5 | 81 | M | L | G | LI | L | 46,XY,t(15;18)(q11.2;q21)[1]/46,XY[2] |  |  |  |  |  |  | + | 1 |
| 6 | 70 | F | HI | P | HI | LI | 46,XX,-1,-2,-4,del(6)(q?),-7,-9,-13,-13,-16,-18,+9mar[1] |  |  |  |  |  |  |  | 1 |
| 7 | 76 | M | HI | P | HI | LI | 47,X,+X,-Y,del(1)(p11p13),add(2)(p11.2),add(3)(q27),der(3)add(3)(p13)add(3)(q27),del(5)(q?),add(7)(p11.2),add(7)(q22),add(10)(p11.2),del(13)(q12q14),add(14)(q22),add(16)(q13),+20,add(21)(p11.2)[17]/46,XY[2] (other 1 cell with 46 chromosomes) |  | + | + |  |  |  |  | 2 |
| 8 | 76 | F | H | P | H | H | 47,X,-X,-1,del(1)(q?),del(4)(q?),add(7)(p11.2),add(9)(p13),add(11)(q23),-13,-15,-16,-16,+add(21)(q22),-22,+7mar[1] (other 2 cells with 46 chromosomes, 5cells with 47 chromosomes, 1 cell with 48 chromosomes, 1 cell with 93 chromosomes, 1 cell with 95 chromosomes) |  |  |  |  | + |  |  | ≧3 |
| 9 | 76 | M | LI | G | HI | LI | 45,X,dic(Y;22)(q12;p11.2),add(11)(q13)[8] (other 1 cell with 45 chromosomes) |  |  |  |  |  | + |  | 2 |
| 10 | 75 | F | H | P | H | H | 63<2n>,X,-X,-3,+4,+6,+7,+7,+7,-8,del(9)(p?),+11,+12,-13,-14,add(14)(q24),-15,-17,+20,+20,+21,+14mar[1] (other 2 cells with 61 chromosomes, 9 cells with 62 chromosomes, 4 cells with 63 chromosomes, 3 cells with 64 chromosomes, 1 cell with 65 chromosomes) |  |  |  |  |  |  |  | ≧3 |
| 11 | 55 | M | H | P | HI | H | 46,XY,t(2;14)(p13;q32),t(3;19)(q27;q13.3)x2[1]/49,s1,+4,+5,+7[6]/49,sdl1,add(4)(q21)[12] (other 1 cell with 48 chromosomes) | + | + |  |  |  |  |  | ≧3 |
| 12 | 35 | M | L | VG | L | L | 46,XY[12] |  |  |  |  |  |  |  | 0 |
| 13 | 76 | F | L | G | LI | L | 47,X,-X,add(1)(p11),add(1)(q21),+3,-6,-7,-8,add(11)(q23),-13,-14,-15,-17,-18,-20,-22,+11mar[1] (other 1 cell with 41 chromosomes, 1 cell with 47 chromosomes, 2 cells with 48 chromosomes, 1 cell with 49 chromosomes, 1 cell with 51 chromosomes) |  |  |  |  |  |  |  | ≧3 |
| 14 | 44 | F | L | VG | L | L | 47,XX,add(4)(q21),del(6)(q?),-13,der(14)t(1;14)(q21;q24),-15,+16,+mar1,+mar2[4]/48,s1,+der(7)t(1;7)(q21;p22),+14,-der(14)t(1;14)[2]/46,XX[14] |  |  |  |  |  |  |  | 2 |
| 15 | 52 | M | L | VG | L | L | 46,XY[20} |  |  |  |  |  |  |  | 0 |
| 16 | 76 | F | HI | P | HI | LI | 45,X,-X,t(3;14)(q27;q32),add(11)(q23)[11]/46,idem,+15[9] | + | + |  |  |  |  |  | 2 |
| 17 | 56 | F | HI | G | LI | LI | 92,XX,-X,add(X)(p11.2),-3,add(4)(q21),add(5)(q31),del(6)(q?)x2,-8,-9,-9,-10,-11,add(16)(q12.1),-17,-17,+18,i(18)(q10)x2,+2r,+6mar[1]/46,XX[4] (other 1 cell with 89 chromosomes, 1 cell with 90 chromosomes, 8 cells with 91 chromosomes, 4 cells with 92 chromosomes, 1 cell with 93 chromosomes) |  |  |  |  |  |  |  | ≧3 |
| 18 | 60 | M | LI | G | LI | LI | 46,XY[1] |  |  |  |  |  |  |  | 0 |
| 19 | 54 | F | H | P | HI | HI | 47,XX,+X,der(1)del(1)(p11p13)ins(1;?)(q21;?)dup(1)(q42q21),add(3)(p21),add(3)(q27),add(5)(q31),del(6)(q?),del(7)(q?),del(9)(p?),del(13)(q12q14),der(13)t(7;13)(q11.2;q14),del(15)(q?)[2]/47,idem,i(8)(q10),+13,-del(13),-21,+mar1[17] (other 1 cell with 47 chromosomes) |  | + |  |  |  |  |  | ≧3 |
| 20 | 67 | M | H | P | HI | LI | 45,X,-Y[4]/46,XY,del(5)(q?)[1]/46,XY,del(20)(q1?)[1]/46,XY[14] |  |  |  |  |  |  |  | ≧3 |
| 21 | 77 | F | LI | G | HI | LI | 48,XX,+5,+12[1]/46,XY[19] |  |  |  |  |  |  |  | 1 |
| 22 | 54 | M | L | VG | L | L | 46,XX,add(2)(p13),del(2)(q?),add(6)(p21),del(6)(q?),+7,der(7;17)(q10;q10),i(18)(q10)[5]/46,idem,add(1)(q21)[12] (other 2 cells with 46 chromosomes 1 cell with 47 chromosomes) |  |  |  |  |  |  |  | ≧3 |
| 23 | 74 | M | H | P | H | H | 49,XY,+Y,+add(Y)(q12),add(1)(q32),add(2)(q21),add(4)(p14),add(6)(p11),del(6)(q?),-10,del(14)(q22),add(16)(q11.2),-18,i(18)(q10),+mar1,+mar2,+mar3[11]/53,s1,+Y,+Y,-add(Y),+7,+13,+mar3[4]/46,XY[2] (other 2 cells with 48 chromosomes, 1 cell with 52 chromosomes) |  |  |  |  |  |  |  | ≧3 |
| 24 | 55 | M | L | VG | L | L | 46,XY[20] |  |  |  |  |  |  |  | 0 |
| 25 | 56 | M | LI | G | LI | HI | 75,XXY,i(1)(q10)x2,+3,-6,del(6)(q?),-9,add(9)(q34),+12,-13,-13,-18,-18,-19,-22,+12mar[1]/46,XY[18] (other 1 cell with 75 chromosomes) |  |  |  |  |  |  |  | 2 |
| 26 | 73 | F | LI | G | LI | LI | 47,XX,+X[3]/46,XX[17] |  |  |  |  |  |  |  | 1 |
| 27 | 75 | M | L | G | LI | L | 65,XXY,i(1)(q10),add(2)(p21)x2,add(3)(q27),-4,-5,-6,+add(7)(q32),-10,+12,-13,-16,-18,-19,+21,+mar1[3]/46,XY[14] (other 1 cell with 64 chromosomes, 1 cell with 66 chromosomes) |  | + |  |  |  |  |  | ≧3 |
| 28 | 61 | F | L | G | LI | L | 48,XX,+X,+11[2]/46,XX[1] |  |  |  |  |  |  |  | 1 |
| 29 | 70 | F | HI | P | HI | HI | 46,XX,der(1)t(1;3)(q42;p21),der(3)t(1;3)t(3;12)(q27;p13),inv(3)(p21q21),t(4;7)(q31;p13),der(12)t(3;12),-19,-21,+mar1,+mar2[8]/46,idem,+add(1)(q21),-der(1)t(1;3)[6]/46,idem,+add(1)(q21),-der(1)t(1;3),add(14)(q32)[1]/46,XX[2] (other 3 cells with 46 chromosomes) | + | + |  |  |  |  |  | ≧3 |
| 30 | 69 | M | H | P | HI | LI | 49,XX,+3,t(8;22)(q24;q11.2),+13,del(15)(q?),+18[4]/46,XY[16] |  |  |  | + |  |  |  | 1 |
| 31 | 73 | F | L | G | LI | L | 91<2n>,XX,+1,+add(1)(q21),+2,+add(3)(q11.2),-5,-5,+i(6)(p10),-8,-8,+11,+11,+12,+12,+13,+13,+14,+16,+17,i(17)(q10)x2,add(18)(q21),+19,+20,+20,+32mar[1]/46,XX[18] (other 1 cell with 123 chromosomes) |  |  |  |  |  |  | + | 2 |
| 32 | 69 | M | LI | G | LI | L | 45,XY,add(1)(p11),t(11;22)(q21;q11.2),add(2)(q11.2),add(3)(q27),add(5)(q35),-6,add(9)(p13),t(14;18)(q32;q21),der(16)t(6:16)(p11;q12.1)[11]/45,idem,-add(3),+add(3)(q27)[8]/46,XY[1] | + | + |  |  | + |  | + | 2 |
| 33 | 80 | M | HI | P | HI | LI | 46,XY,add(1)(q21)[1]/84<3n>,XXY,+X,+Y,+add(1),+2,+3,-4,+5,+6,-7,+8,+10,+11,+13,-16,-17,+21,+7mar[1]/46,XY,add(1)(p11),add(18)(q11.2)[1]/46,XY[17] |  |  |  |  |  |  |  | ≧3 |
| 34 | 69 | F | HI | P | HI | LI | 92<3n>,X,-X,-X,add(1)(q11)x2,i(1)(q10),add(3)(p13),add(3)(p21),add(3)(q11.2),-4,add(4)(q21),-5,del(5)(q?),+add(6)(q21),+del(6)(q?),+add(7)(q11.2),+i(7)(q10),-9,add(10)(q22),-13,-14,add(15)(p11.2),+16,+add(17)(p11.2),+19,+20,+21,+21mar[1]/46,XX[1] (other 1 cell with 83 chromosomes, 2 cells with 89 chromosomes, 2 cells with 90 chromosomes, 1 cell with 91 chromosomes, 2 cells with 92 chromosomes, 6 cells with 93 chromosomes, 3 cells with 94 chromosomes, 1 cell with 95 chromosomes) |  |  |  |  |  |  |  | ≧3 |
| 35 | 85 | M | HI | P | HI | LI | 47,X,-Y,add(1)(q42),+6,+dup(7)(q22q32),del(8)(p11.2),del(9)(p?),der(19)t(1;19)(q25;q13.3)[10]/48,idem,+12[1]/46,XY[9] |  |  | + |  |  |  |  | 2 |
| 36 | 69 | M | H | P | H | H | 45,X,-Y,add(3)(q21),del(6)(q15),der(14)?t(3;14)(q27;q32),del(16)(q?),add(20)(p13)[3]/45,s1,add(2)(p11.2)[6]/45,s1,+add(3)(q12),-add(3),+add(14)(q32),-der(14)?t(3;14)[2]/46,XY[6] (other 1 cell with 45 chromosomes, 2 cells with 46 chromosomes) | + | + |  |  |  |  |  | ≧3 |
| 37 | 82 | F | LI | P | HI | LI | 53,XX,+X,add(1)(q32),der(3)add(3)(p25)add(3)(q21),add(5)(q13),del(6)(q12),-8,add(10)(q22),-13,-14,+16,-17,+18,+der(18)t(11;18)(q13;q23),+19,+der(?)t(?;14)(?;q11.2),+mar1,+mar2,+mar3,+mar4x2[1]/53,s1,-der(3)add(3)add(3),+der(3)add(3)add(3)(q21),-13,-16,-19,-mar4x2,+6mar[1]/46,XX[10] (other 1 cell with 50 chromosomes, 1 cell with 52 chromosomes, 2 cells with 53 chromosomes, 2 cells with 54 chromosomes, 2 cells with 55 chromosomes) |  |  |  |  |  | + |  | ≧3 |
| 38 | 59 | M | L | VG | L | L | 46,XY[1] |  |  |  |  |  |  |  | 0 |
| 39 | 74 | M | L | G | LI | L | 100,XXYY,?i(1)(q10),+add(3)(q11.2)x2,add(3)(q27)x2,add(4)(q31)x2,+5,-6,-6,-6,add(6)(q13),-10,-13,-13,-14,-14,-14,add(14)(q32),-16,-16,+19,+20,+14mar[1]/46,XY[7] (other 1 cell with 96 chromosomes, 1 cell with 98 chromosomes) | + | + |  |  |  |  |  | ≧3 |
| 40 | 76 | F | H | P | H | H | 48,XX,add(2)(q11.2),add(3)(p11),add(3)(p11),add(4)(q21),-14,+15,+mar1,+mar2[2]/46,XX[18] |  |  |  |  |  |  |  | 1 |
| 41 | 71 | M | LI | G | LI | LI | 48,XY,add(1)(q32),+3,+add(3)(p13),del(15)(q?),der(17;22)(q10;q10),+18,add(19)(q13.1)[7]/46,XY[13] |  |  |  |  |  |  |  | 1 |
| 42 | 63 | F | H | P | HI | LI | 47,XX,t(3;22)(q27;q11.2),+7,t(8;14)(q24;q32)[3]/46,XX[17] | + | + |  | + |  |  |  | 1 |
| 43 | 75 | M | HI | P | HI | L | 47,XY,+15[4]/46,XY[16] |  |  |  |  |  |  |  | 1 |
| 44 | 70 | M | L | G | LI | L | 83,XX,-Y,-Y,-1,-1,-2,-2,-3,add(3)(q21),-5,-6,-6,del(6)(q?),+7,-8,-9,-9,-10,-10,-11,add(11)(p15),-12,-13,-14,-14,-16,-17,add(18)(p11.2)x2,-19,-19,-22,+15mar[1]/46,XY[19] |  |  |  |  |  |  |  | 1 |
| 45 | 60 | F | H | P | HI | H | 48,XX,add(1)(q44),der(1)add(1)(p32)add(1)(q32),add(3)(q27),add(7)(q22),-16,+20,+21,+mar1[18] (other 1 cell with 49 chromosomes, 1 cell with 50 chromosomes) |  | + | + |  |  |  |  | ≧3 |
| 46 | 66 | M | H | P | HI | LI | 45,X,-Y[4]/50,XY,+3,add(11)(q23),der(13)t(1;13)(q12;p13),+14,+18,add(21)(p11.2),+mar1[4]/46,XY[12] |  |  |  |  |  |  |  | 2 |
| 47 | 77 | F | HI | P | H | LI | 47,XX,add(1)(q21),del(2)(q?),-5,-5,-6,-8,-9,-9,-10,-11,-13,-14,-14,-15,-18,-22,+15mar[1] |  |  |  |  |  |  |  | 1 |
| 48 | 65 | M | LI | G | LI | L | 48,XY,+5,?t(8;14;18;13)(q24;q32;q21;q32),+12,del(13)(q?)[17]/46,XY[3] | + |  |  | + |  |  | + | 1 |
| 49 | 83 | M | HI | P | HI | L | 45,X,-Y,inv(9)(p12q13)[16]/46,XY,inv(9)(p12q13)[4] |  |  |  |  |  |  |  | 0 |
| 50 | 57 | M | HI | G | HI | H | 46,XY |  |  |  |  |  |  |  | 0 |
| 51 | 74 | F | HI | P | HI | LI | 48,XX,+X,add(1)(p36.1),t(2;8)(p12;q24),+7,add(13)(p11.2),t(14;18)(q32;q21)[18]/49,idem,+12[2] | + |  |  | + |  |  | + | 2 |
| 52 | 53 | M | L | VG | L | L | 47,X,+X,-Y,del(1)(p?),add(2)(p11.2),+add(3)(p13),add(6)(p21),del(6)(q?),add(7)(p11.2),add(7)(q32),add(9)(p13),add(13)(p11.2),add(14)(q24),add(14)(q32),i(15)(q10),add(16)(q12.1),-17,+mar1[10]/46,XY[1] (other 4 cells with 46 chromosomes, 5 cells with 47 chromosomes) | + |  |  |  | + |  |  | ≧3 |
| 53 | 51 | M | LI | G | LI | L | 50,XY,add(1)(q32),+3,+add(3)(q21),del(6)(q?),del(9)(q?),+13,+18,add(18)(p11.2)x2[6]/49,idem,-Y[8]/46,XY[2] (other 1 cell with 41chromosomes, 2 cells with 50 chromosomes) |  |  |  |  |  |  |  | ≧3 |
| 54 | 73 | M | HI | P | HI | HI | 49,XY,add(1)(q11),t(4;12)(q21;p13),add(6)(p11),i(6)(p10),+7,der(8)add(8)(p11.2)t(1;8)(q11;q24),+der(12)t(4;12),t(12;20)(q13;p13),-13,add(18)(q21),+mar1x2[13]/46,XY[7] |  |  |  | + |  |  | + | 1 |
| 55 | 79 | F | LI | G | HI | LI | 92,XXXX,-1,-1,t(2;3)(p12;q27)x2,+6,add(6)(q13)x3,-10,+der(?)t(?;1)(?;p13)x2[1]/46,XX[16] (other 1 cell with 92 chromosomes, 1 cell with 94 chromosomes) |  | + |  |  |  |  |  | 1 |
| 56 | 49 | F | L | VG | L | L | 48,XX,+add(X)(q22),der(4)t(4;12)(q31;q13),der(4;?)t(4;?)(p15;?)t(?;12)(?;q13),add(7)(q22),add(9)(p13),+21[6]/46,XX[14] |  |  | + |  | + |  |  | 1 |
| 57 | 79 | M | H | P | H | H | 100<2n>,XY,+X,+Y,+1,+add(1)(p11),+2,+3,+3,-4,+6,+add(6)(q13),-7,+add(8)(p11.2),-10,add(11)(p11.2)x2,-12,-13,-13,+14,-17,add(18)(q21),+20,+20,+46mar[1] (other 1 cell with 99 chromosomes, 1 cell with 100 chromosomes) |  |  |  |  |  |  | + | 1 |
| 58 | 55 | F | L | VG | L | L | 50,XX,-2,-8,-9,+11,-17,+7mar[1] |  |  |  |  |  |  |  | 1 |
| 59 | 38 | M | L | VG | L | L | 57,X,-Y,add(1)(q21),+5,+6,+7,+9,+11,+12,add(15)(q22),+19,+mar1,+mar2,+mar3,+mar4,+mar5[4]/46,XY[14] (other 1 cell with 50 chromosomes, 1 cell with 55 chromosomes) |  |  |  |  |  |  |  | ≧3 |
| 60 | 64 | M | HI | P | HI | LI | 47,XX,+X,add(1)(p36.1),-6,+7,t(14;18)(q32;q21)[6]/47,idem,del(5)(q?)[11]/46,XY[3] | + |  |  |  |  |  | + | 2 |
| 61 | 78 | M | H | P | H | LI | 75<2n>,X,-Y,+1,-4,-4,-6,-13,-14,-14,-17,-18,+19,+36mar[1] (other 1 cell with 72 chromosomes, 1 cell with 73 chromosomes, 2 cells with 74 chromosomes, 3 cells with 75 chromosomes, 1 cell with 76 chromosomes, 1 cell with 79 chromosomes) |  |  |  |  |  |  |  | ≧3 |
| 62 | 34 | M | LI | G | L | L | 86,XXYY,i(1)(q10)x2,-4,-4,-8,?t(8;14)(q24;q32),-10,add(13)(q14)x2,-14,-14,-15,-15,-16,-17,-17,+mar1,+mar2,+mar3,+mar4,+mar5[2]/46,XY[1] (other 1 cell with 84 chromosomes, 1 cell with 85 chromosomes, 1 cell with 86 chromosomes) | + |  |  | + |  |  |  | ≧3 |
| 63 | 72 | F | H | P | H | HI | 46,XX |  |  |  |  |  |  |  | 0 |
| 64 | 83 | F | L | G | LI | L | 46,XX,t(7;11)(q32;q13)[2]/46,XX[1] |  |  |  |  |  | + |  | 1 |
| 65 | 67 | F | H | P | HI | LI | 97,XXXX,-3,i(6)(p10)x2,-13,-13,?t(14;18)(q32;q21)x2,-16,-18,+10mar[1]/46,XX[1] (other 1 cell with 98 chromosomes) | + |  |  |  |  |  | + | 2 |
| 66 | 62 | M | HI | P | HI | LI | 47,X,-Y,+3,del(6)(q?),add(7)(q22),del(7)(q?),add(8)(q22),add(9)(p13),-10,-10,add(11)(p11.2),add(11)(q23),add(12)(q22),add(15)(p11.2),+17,-22,+mar1,+mar2,+mar3[5]/46,XY[1] (other 2 cells with 46 chromosomes, 3 cells with 47 chromosomes) |  |  | + |  | + |  |  | ≧3 |
| 67 | 73 | M | HI | P | HI | HI | 46,XY |  |  |  |  |  |  |  | 0 |
| 68 | 54 | M | L | VG | L | L | 46,XY |  |  |  |  |  |  |  | 0 |
| 69 | 66 | M | LI | G | HI | LI | 47,XY,+X[1]/46,XY[19] |  |  |  |  |  |  |  | 1 |
| 70 | 48 | M | LI | G | LI | L | 86,XX,-Y,-Y,-3,-4,+6,del(6)(q?)x3,+8,-10,-11,der(15;17)(q10;q10)x2[2]/46,XY[18] |  |  |  |  |  |  |  | 1 |
| 71 | 65 | M | HI | P | HI | LI | 82<3n>,XX,+X,-Y,trp(1)(q21q32),-2,add(2)(q31),?t(3;14)(q27;q32),+6,del(6)(q?)x2,-9,-10,+11,del(11)(q?)x2,-12,-13,-15,-15,-16,-17,add(18)(q21),+20,+20,+21,+21,-22,+17mar[1]/46,XY[1] (other 2 cells with 80 chromosomes, 3 cells with 81 chromosomes, 3 cells with 82 chromosomes, 6 cells with 83 chromosomes, 1 cell with 85 chromosomes) | + | + |  |  |  |  | + | ≧3 |
| 72 | 67 | F | H | P | H | H | 49,X,-X,-2,t(14;18)(q32;q21),+r1,+mar1,+mar2,+mar3,+mar4[4]/49,s1,ins(1;?),(q21;?)[6]/46,XX[1] (other 5 cells with 48 chromosomes, 4 cells with 49 chromosomes) | + |  |  |  |  |  | + | ≧3 |
| 73 | 59 | F | H | P | H | H | 47,XX,-4,t(8;14)(q24;q32),-13,+add(14)(q32),der(16)t(16;17)(q24;q11.2),-17,der(20)t(1;20)(q12;q13.3),-22,+r1,+mar1,+mar2,+mar3[20] | + |  |  | + |  |  |  | 1 |
| 74 | 53 | M | L | VG | L | L | 57,XY,+X,der(1)(qter→q12::p36.1→qter),+2,+3,+5,+7,+11,+11,+12,-14,t(14;18)(q32;q21),-15,-15,-16,-17,+18,+7mar[1]/46,XY[19] | + |  |  |  |  |  | + | 1 |
| 75 | 78 | M | L | G | LI | L | 47,XY,add(1)(p36.1),+3,-7,+8,del(20)(q11.2q13.3)[2] |  |  |  |  |  |  |  | 1 |
| 76 | 69 | M | HI | P | HI | LI | 46,XY[1] |  |  |  |  |  |  |  | 0 |
| 77 | 71 | F | LI | G | LI | L | 48,XX,-4,+7,t(8;22)(q24;q11.2),inv(9)(p12q13),+11,t(14;18)(q32;q21),add(17)(p11.2),add(21)(p11.2),+mar1[6]/48,s1,-add(17),+mar2[4]/46,XX,inv(9)(p12q13)[9] (other 1 cell with 49 chromosomes) | + |  |  | + |  |  | + | ≧3 |
| 78 | 58 | F | H | P | HI | LI | 48,XX,add(11)(q13),+12,add(17)(q11.2),+mar1[5]/46,XX[11] |  |  |  |  |  | + |  | 1 |
| 79 | 67 | F | LI | G | LI | LI | 46,XX,t(3;16)(q21;p13.3)[1]/46,XX[19] |  |  |  |  |  |  |  | 1 |
| 80 | 74 | M | LI | G | LI | LI | 46,XY[2] |  |  |  |  |  |  |  | 0 |
| 81 | 73 | F | HI | P | HI | HI | 46,XX,der(3)add(3)(p21)add(3)(q27),add(4)(q21),add(5)(p13),inv(9)(p12q13),t(12;19)(q13;p13),-16,add(16)(p11.2),-17,-18,-19,+mar1,+mar2,+mar3,+mar4[2]/46,XX,inv(9)(p12q13)[3] (other 2 cells with 45 chromosomes) |  | + |  |  |  |  |  | ≧3 |
| 82 | 76 | M | HI | P | HI | LI | 46,XY[1] |  |  |  |  |  |  |  | 0 |
| 83 | 77 | M | H | P | H | H | 45,X,-Y[8]/46,XY[12] |  |  |  |  |  |  |  | 1 |
| 84 | 72 | M | L | G | LI | LI | 86~89,XX,-Y,-Y,add(1)(p34.1),add(2)(q31),+3,+3,add(6)(p21.3)x2,-7,-10,add(11)(q11)x2,add(11)(q23)x2,-13,-14,add(14)(q32),+16,add(17)(q25),add(18)(p11.2)x2,-21,-22,+mar1x2[cp29] (6 cells with 86 chromosomes, 3 cells with 87 chromosomes, 4 cells with 88 chromosomes, 7 cells with 89 chromosomes) | + |  |  |  |  |  |  | ≧3 |
| 85 | 67 | M | L | G | LI | L | 46,XY,del(1)(p?32),inc[1] |  |  |  |  |  |  |  | 1 |
| 86 | 83 | F | HI | P | H | H | 49~51,X,-X,t(1;14)(q21;q32),+6,i(6)(p10),+7+8,+10,add(10)(q22),t(14;18)(q32;q21),add(17)(q25),+18,der(18)t(14;18),+mar1[cp14]/62~80,XX,-X,+1,t(1;14)x2,-2,+3,i(6),t(14;18),+18,+20,+20[cp6] (1 cell with 49 chromosomes, 9 cells with 50 chromosomes, 4 cells with 51 chromosomes, 1 cell with 62 chromosomes, 1 cell with 63 chromosomes, 2 cells with 67 chromosomes, 1 cell with 78 chromosomes, 1 cell with 80 chromosomes) | + |  |  |  |  |  | + | ≧3 |
| 87 | 79 | M | L | G | LI | LI | 46,XY[3] |  |  |  |  |  |  |  | 0 |
| 88 | 36 | M | L | VG | L | L | 46,XY,inv(11)(p15q23)[5]/47,s1,+16[8]/47,sdl1,add(9)(p11)[2]/46,XY[5] |  |  |  |  |  |  |  | ≧3 |
| 89 | 44 | M | L | G | LI | L | 48,Y,add(X)(p11.2),add(1)(q21),+11,t(14;19)(q32;q13.1),add(18)(q21),+mar1[17]/46,XY[2] (other 1 cell with 47 chromosomes) | + |  |  |  |  |  | + | 2 |
| 90 | 70 | F | L | G | LI | L | 46,XY |  |  |  |  |  |  |  | 0 |
| 91 | 78 | F | HI | P | H | H | 93,XX,-X,-X,-1,-2,-4,+add(9)(q34)x3,-10,-11,-15,-17,add(17)(p11.2),add(17)(p11.2),del(17)(p11.2),i(18)(q10)x2,-22,-22,-22,-22,+der(?)t(?;1)(?;q21),+der(?)t(?;1)(?;q21),+der(?)t(?;1)(?;q21),+9mar[1]/46,XX[10] (other 1 cell with 88 chromosomes, 1 cell with 89 chromosomes, 2 cells with 91 chromosomes, 2 cells with 92 chromosomes, 2 cells with 94 chromosomes, 1 cell with 95 chromosomes) |  |  |  |  |  |  |  | ≧3 |
| 92 | 48 | F | HI | P | HI | HI | 48,XX,add(1)(p36.1),-2,del(2)(p?),add(3)(q11.2),del(3)(p?),add(4)(p11),del(5)(q?),-8,-9,add(11)(q13),-14,-17,del(20)(q1?),+der(?)t(?;1)(?;q21),+mar1,+mar2,+mar3,+mar4,+mar5,+mar6[20] |  |  |  |  |  | + |  | 1 |
| 93 | 79 | F | LI | G | HI | L | 45,XX,add(2)(p13),add(2)(q33),-6,add(6)(p11),add(8)(p11.2),-10,del(11)(p?),add(12)(q13),-13,t(14;18)(q32;q21),add(16)(p11.2),-17,der(20)t(11;20)(q13;q11.2),+mar1,+mar2,+mar3[11]/46,XX[6] (other 3 cells with 45 chromosomes) | + |  |  |  |  | + | + | ≧3 |
| 94 | 80 | M | H | P | H | H | 82,YY,add(X)(q22)x2,-1,-1,add(1)(p11),add(1)(p11),-2,add(3)(q12),add(3)(q21),-4,-5,add(5)(p11)x2,-6,add(6)(q13),-7,-8,-9,-10,add(10)(q22),-12,-14,-14,add(14)(q32)x2,add(15)(p11.2),-16,-18,add(18)(q21)x2,+21,+21,-22,+4mar[1]/46,XY[6] (other 1 cell with 81 chromosomes, 3 cells with 82 chromosomes, 3 cells with 83 chromosomes, 2 cells with 84 chromosomes, 1 cell with 85 chromosomes, 1cell with 88 chromosomes, 1 cell with 89 chromosomes, 1 cell with 90 chromosomes) | + |  |  |  |  |  | + | ≧3 |
| 95 | 84 | F | HI | P | H | HI | 44,X,-X,-13[17]/46,XX[3] |  |  |  |  |  |  |  | 1 |
| 96 | 80 | F | H | P | H | H | 90,XXXX,-1,-2,-2,-3,t(3;22)(q27;q11.2)x2,add(6)(q21),-7,+8,-9,add(9)(p13),+12,-14,der(14)t(1;14)(q21;q24),t(14;18)(q32;q21),-15,-16,der(18)t(14;18),+20,+mar1,+mar2,+mar3,+mar4[2]/46,XX[15] (other 3 cells with 90 chromosomes) | + | + |  |  | + |  | + | ≧3 |
| 97 | 81 | F | H | P | H | HI | 47,X,add(X)(q22),add(7)(q22),+17[20] |  |  | + |  |  |  |  | 1 |
| 98 | 73 | M | HI | P | HI | L | 91~96,XXYY,add(1)(p13),i(1)(q10),+3,-7,add(11)(q?25),+18,+mar1,+mar2,+mar3,+mar4,+mar5x2,inc[cp3]/46,XY,inc[17] (17 cells with 46 chromosomes (46,XY), 1 cell with 91 chromosomes, 1 cell with 95 chromosomes, 1 cell with 96 chromosomes) |  |  |  |  |  |  |  | ≧3 |
| 99 | 47 | F | LI | G | LI | LI | 45,XX,?der(1;14)(q10;q10)[2] |  |  |  |  |  |  |  | 1 |
| 100 | 76 | F | L | VG | LI | L | 46,XX[1] |  |  |  |  |  |  |  | 0 |
| 101 | 85 | M | H | P | H | H | 66,XY,-X,-1,-2,-4,-5,del(6)(q?),-7,add(7)(p11.2),+8,+8,+8,-9,-9,+13,-16,-17,-18,+21,+der(1;7)(q10;p10)x2,+mar1[13] (other 1 cell with 66 chromosomes, 6 cells with 67 chromosomes) |  |  |  |  |  |  |  | ≧3 |
| 102 | 78 | F | H | P | H | H | 48,XX,+X,add(1)(p34),t(1;8)(q21;q11.2),der(6)add(6)(p21)add(6)(q21),der(9)t(9;12)(p13;p13)ins(9;?)(p13;?),add(11)(q23),+18[9]/46,XX[10] (other 1 cell with 48 chromosomes) |  |  |  |  | + |  |  | 2 |
| 103 | 84 | F | LI | G | HI | LI | 45,X,-X[4] |  |  |  |  |  |  |  | 1 |
| 104 | 69 | F | HI | P | HI | LI | 47,XX,del(1)(q21),dup(1)(q21q32),der(7)t(1;7)(?;p15),+del(9)(q12),der(13)t(13;15)(p11.2;q?),t(14;17)(q22;q21)[1]/46,XX[10] (other 1 cell with 45 chromosomes, 1 cell with 47 chromosomes, 1 cell with 50 chromosomes) |  |  |  |  |  |  |  | ≧3 |
| 105 | 79 | F | L | G | LI | L | 45,XX,-4,-5,-6,-7,add(9)(p13),-14,-14,-18,del(20)(q1?),+6mar[1] |  |  |  |  | + |  |  | 1 |
| 106 | 43 | F | L | VG | L | L | 41,X,-X,add(1)(p36.1),-2,add(7)(q11.2),-8,-10,-13,-14,-18,add(18)(q21),add(19)(q13.1),-20,-20,-21,-22,+der(?)t(?;1)(?;q12),+5mar[1]/46,XX[2] (other 1 cell with 41 chromosomes) |  |  |  |  |  |  | + | 2 |
| 107 | 76 | F | H | P | H | H | 46,XX,?inv(2)(p11.2q14.2)[1] |  |  |  |  |  |  |  | 1 |
| 108 | 76 | M | H | P | H | H | 46,XY,inc[6] |  |  |  |  |  |  |  | 0 |
| 109 | 82 | M | L | VG | LI | L | 51,XY,dup(1)(q12q32),+3,-4,+5,+del(6)(q?),+7,del(9)(p?),-10,del(12)(p?),+3mar[1] |  |  |  |  |  |  |  | 1 |
| 110 | 64 | M | LI | G | LI | LI | 47,-Y,add(X)(q22),add(3)(q11.2),add(4)(q31),add(5)(p11),add(6)(p11),-8,+add(9)(p13),add(10)(p11.2),add(11)(p11.2),add(12)(p11.2),der(13;15)(q10;q10),-15,-16,del(17)(p11.2),+18,+18,+18,-19,-21,+mar1,+mar2,+mar3,+mar4[2]/48,idem,+18[5] (other 1 cell with 43 chromosomes, 1 cell with 44 chromosomes, 1 cell with 45 chromosomes, 1 cell with 46 chromosomes, 4 cells with 47 chromosomes, 4 cells with 48 chromosomes, 1 cell with 49 chromosomes) |  |  |  |  | + |  |  | ≧3 |
| 111 | 79 | F | H | P | H | HI | 45,X,add(X)(q22),-4,add(5)(p11),-6,-7,-8,add(9)(p13),add(17)(p11.2),-22,+4mar[1]/46,XX[3] (other 3 cells with 45 chromosomes) |  |  |  |  | + |  |  | ≧3 |
| 112 | 76 | M | LI | G | HI | L | 45,X,-Y[4]/46,XY,t(1;13)(q11;p11.2)[1]/46,XY[15] |  |  |  |  |  |  |  | 2 |
| 113 | 63 | M | HI | P | LI | LI | 45,X,-Y[14]/46,idem,+10[1]/45,idem,-8,-11,+2mar[1] |  |  |  |  |  |  |  | ≧3 |
| 114 | 59 | F | L | G | L | L | 86,XXXX,-1,ins(1;?)(q21;?),-3,-4,-4,-5,del(6)(q?),+7,-8,+9,+10,+12,-13,-15,-15,add(17)(p11.2)x2,-18,-21,-22,+2mar[1]/46,XX[18] (other 1 cell with 85 chromosomes) |  |  |  |  |  |  |  | 2 |
| 115 | 55 | M | L | G | LI | LI | 48,XY,+3,+3,add(9)(q11),-15,add(19)(p11),der(20)t(15;20)(q15;q13.1),+mar1[18]/46,XY[2] |  |  |  |  |  |  |  | 1 |
| 116 | 70 | F | L | G | LI | L | 48,X,-X,+add(3)(p21),+7,add(10)(q22),+18[3]/49,idem,+der(3)t(1;3)(q21;p25)[3]/46,XX[13] (other 1 cell with 49 chromosomes) |  |  |  |  |  |  |  | 2 |
| 117 | 71 | F | HI | P | HI | HI | 87,XXXX,-1,add(1)(q21)x2,add(1)(q21),+2,add(2)(q11.2)x3,add(3)(q21),-4,-6,-6,add(6)(p21)x2,+7,+7,-8,-11,-13,-14,-14,-15,add(17)(q25),-19,-20,+4mar[1]/46,XX[5] (other 1 cell with 84 chromosomes, 1 cell with 86 chromosomes, 2 cells with 87 chromosomes, 1 cell with 88 chromosomes, 3 cells with 89 chromosomes, 1 cell with 91 chromosomes, 1 cell with 92 chromosomes) |  |  |  |  |  |  |  | ≧3 |
| 118 | 75 | F | LI | G | LI | L | 84,XXX,-X,-2,-2,+3,-5,-5,-5,-5,-6,-6,-8,-10,-10,-12,-14,-18,-19,-19,-21,-22,+10mar[8]/46,XX[2] |  |  |  |  |  |  |  | 1 |
| 119 | 70 | M | H | P | HI | LI | 86<2n>,XY,-3,+5,+7,+18,+20,+21,+22,+34mar[1]/46,XY[1] |  |  |  |  |  |  |  | 1 |
| 120 | 62 | M | H | P | HI | LI | 92,XXY,-Y,-3,-3,-4,del(6)(q?),+7,+7,-8,der(8)t(8;13)(p21;q12)x2,add(9)(p11),add(9)(p11),-10,del(10)(q?),del(11)(p?),-13,-13,-13,-13,der(14)t(14;18)(q32;q21),t(14;18),del(17)(p11.2)x2,-18,-21,-21,-21,-22,+13mar,32dmin[1] (other 2 cells with 85 chromosomes, 1 cell with 88 chromosomes, 1 cell with 89 chromosomes, 1 cell with 90 chromosomes, 1 cell with 91 chromosomes, 4 cells with 92 chromosomes, 5 cells with 93 chromosomes 4 cells with 94 chromosomes) | + |  |  |  |  |  | + | ≧3 |
