# Supplementary material for: Chromosomal abnormality variation detected by G‐banding is associated with prognosis of diffuse large B‐cell lymphoma treated by R‐CHOP‐based therapy
Source: Cancer Med. 2018 Feb 23;7(3):655–64. doi: 10.1002/cam4.1342 (PMC5852349; doi:10.1002/cam4.1342)
Supplement: Supplementary file 5 [file CAM4-7-655-s005.docx]

**Supplementary Legend**

**Supplementary Table 1.**

Clinical features, chromosomal abnormalities and number of chromosomal abnormality variations (CAVs) in 120 DLBCL patients with available metaphase spreads.

**Supplementary Figure 1.**

Overall survival of patients classified by R-IPI, NCCN-IPI, and KPI. L: low risk, LI: low-intermediate risk, HI: high-intermediate risk, H: high risk. R-IPI-defined poor patients, NCCN-IPI-defined high risk patients and KPI-defined high risk patients had poor survival rates, confirming the prognostic significance of each indice within the patients in our study.

**Supplementary Figure 2.**

Patient cohort selection.

**Supplementary Figure 3.**

Overall survival (A) and progression-free survival (B) of patients with and without available metaphase spreads. Neither OS nor PFS was significantly differed between patients with and without available metaphase spreads.
